# Establishing a rabbit model of malignant esophagostenosis using the endoscopic implantation technique for studies on stent innovation

**DOI:** 10.1186/1479-5876-12-40

**Published:** 2014-02-10

**Authors:** Jin Huang, Jinquan Shuang, Guanyin Xiong, Xiang Wang, Yin Zhang, Xiaowei Tang, Zhining Fan, Yingzhou Shen, Hanming Song, Zhi Liu

**Affiliations:** 1 Digestive Medical Center, The Second Affiliated Hospital of Nanjing Medical University, No 121 Jiang Jiayuan, Xiaguan District, Nanjing, Jiangsu Province, 210011, China; 2 Division of Digestive Diseases, Renming Hospital of Ma Anshan, No 45 Hubei Road, Huashan District, Ma Anshan City, Anhui Province, 243000, China

**Keywords:** Esophageal squamous cell carcinoma, Animal model, Endoscopic surgical procedure, Rabbit, Stent therapy

## Abstract

**Background:**

Stents are recommended in patients with dysphagia caused by esophageal stricture, but an ideal stent does not currently exist. Thus, studies on new esophageal stents are necessary, and suitable animal models are desperately needed for these studies. The aim of this study was to establish a model of malignant esophageal stricture in rabbit for studies on stent innovation.

**Methods:**

A total of 38 New Zealand white rabbits were used in this study. Using the endoscopic submucosal injection technique, VX2 fragments were inoculated into the submucosal layer of the rabbit thoracic esophagus, and an endoscopic follow-up was subsequently performed to observe the tumor development and progression. The self-expandable metal stents were randomly deployed in rabbits with severe esophageal stricture to investigate the safety and feasibility of the animal models for stenting.

**Results:**

An endoscopic implantation procedure for VX2 tumors was completed in 34/38 rabbits, and tumor development was confirmed in 30/34 animals. The success rate of the endoscopic implantation and tumor development were 89.4% (95% CI, 79.6% to 99.2%) and 88.2% (95% CI, 76.9% to 99.5%) respectively. During the endoscopic follow-up period, severe esophageal stricture occurred in 22/30 rabbits with a rate of 73.3% (95% CI, 57.5% to 89.1%), and 12/22 models received stent placement. During and after stent implantation, no severe stent-related complication or mortality occurred in the animal models. The rabbits that received stent placement survived longer than those without stent implantation (the mean survival time: 53.9 days versus 40.3 days, *P* = 0.016).

**Conclusion:**

The endoscopic method is a safe and effective method for establishing a malignant esophagostenosis model in rabbits. This model can simulate the human body environment for stent deployment and is an excellent tool for the study of stent innovation for the treatment of esophageal cancer.

## Introduction

Esophageal cancer (EC) is the sixth leading cause of cancer-related mortality and the eighth most common cancer worldwide [1,2]. Treatment of esophageal carcinoma remains challenging but is best approached using a multidisciplinary team. Stents are recommended in patients with dysphagia caused by esophageal stricture, and an optimal stent should have features, such as being easy to deploy, staying in place, reducing tumor ingrowth or overgrowth, and causing minimal to no discomfort [3-5]. However, the ideal stent does not currently exist, and studies on new esophageal stents are necessary and significant. Thus, suitable animal models are desperately needed for these studies.

Theoretically, a good animal model of EC for stenting should be able to mimic the esophagostenosis features of the human disease and be sufficiently large to perform minimally invasive procedures designed for humans. However, there is currently a severe lack of an excellent animal model of EC to study stents [6-9].

The VX2 tumor model, which was originally proposed by Shope and Hurst [10] in 1933, has been developed in the organs of rabbits, including the liver, kidney, lung, head and neck [11-14]. Although the rabbit model belongs to moderate-to-large-sized models and has been used to study EC in relevant research, there have been no reports on establishment of a rabbit model on malignant esophagostenosis, which is available for stent implantation.

Here, we introduce an endoscopic method for the development of a rabbit model of malignant esophagostenosis, and further investigate the safety and feasibility of stent implantation in this model. This is the first study where rabbits with esophageal malignant tumors were used for stent development procedures.

## Material and methods

### Animals and tumors

Male or female New Zealand White rabbits weighing between 2.5 and 3.0 kg were obtained from The Jiangsu Agricultural Academy of Science in China and food and water were provided ad libitum. All experimental procedures were approved by the Animal Care and Use Subcommittee at Nanjing Medical University.

### Donor rabbits and implantation preparation

Rabbits with hind limb tumors (donor) were used to propagate and maintain VX2 tumors. Approximately 2 to 3 pieces of thawed VX2 tumor tissue fragments (approximately 0.5 mm^3^) that, were previously stored in liquid nitrogen were injected deep into the hind limb of gluteal muscles of rabbits anesthetized using an intramuscular injection of 35-mg/kg pentobarbital sodium (Sigma Chemical Co., St. Louis, MO, USA). Three weeks after implantation, the animals were sacrificed and the hind limb tumors were harvested and immediately processed. All VX2 tumors were cleaned from the surrounding tissue and gross necrotic portions of the tumors were removed.

The collected tumors were cut into small pieces (approximately 0.5 mm^3^) and preserved in saline for fragment implantation. approximately 0.3 ml saline solution with 4 pieces of tumor fragments were placed in a 2-ml syringe on ice until they were injected into the recipient rabbit’s esophagus.

### Endoscopic implantation of VX2 tumors

The rabbits were first anesthetized after 24 h of fasting with free access to water and then placed in the left lateral decubitus position. An endoscope (Olympus GIFXP260, Japan) was inserted into the thoracic esophagus. Using a fine endoscopic needle (the inner core of an endoscopic needle, Olympus, MAJ-68, 23-gauge), 0.5 ml saline was injected into the submucosal layer to elevate the mucosa layer. Next, a puncture was made using a modified endoscopic needle (Olympus, MAJ-68, with an oblique cut in the front, approximately 20-gauge) across the mucosa into the submucosal layer and the success of the puncture was confirmed by further elevation of the mucosa when saline was injected. About 0.3 ml saline containing 4 tumor pieces was then injected into the submucosal layer of rabbits esophagus (an additional movie file shows this in more detail, see Additional file 1). A second 0.3 ml saline was injected to rinse the needles and to collect the remaining pieces. After implantation, the rabbits were given an auricular vein infusion and fasted for 24 h.

### Endoscopic follow-up

Endoscopic examinations were performed once a week after implantation to observe tumor growth and the degree of esophageal stenosis. Tumor development was determined using endoscopy and biopsy. Three grades were used to evaluate the esophageal stenosis under endoscopy: mild stenosis for tumors less than or equal to 1/3 of the lumen diameter, moderate stenosis for tumors more than 1/3 but less than or equal to 2/3 of the lumen diameter, and severe stenosis for tumors more than 2/3 of the lumen diameter.

### Stent implantation

**Figure 1:**
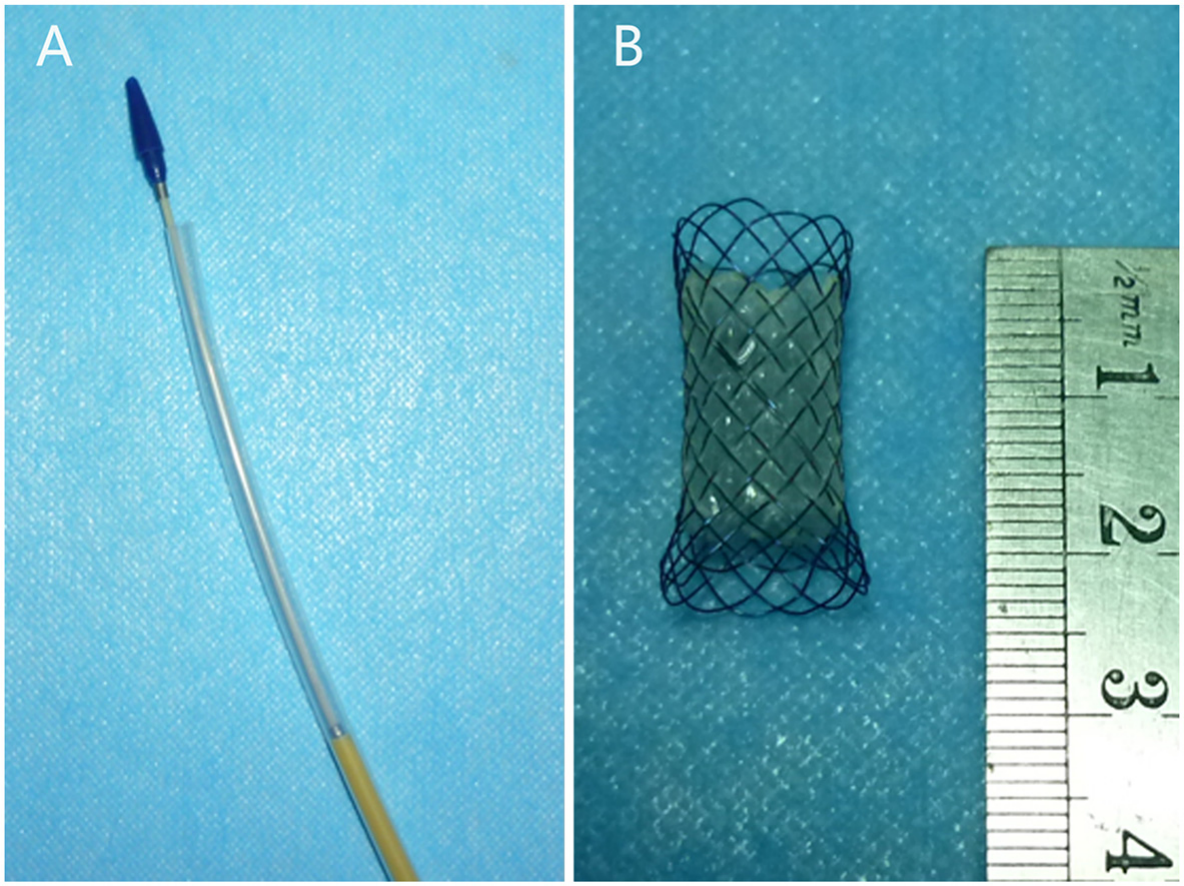
**Stent and its delivery system. (A)** The delivery system for the stent. **(B)** The self-expandable metal stent is 8 mm in diameter and 25 mm in length, and its partial length is covered with a silicone membrane.

**Figure 2:**
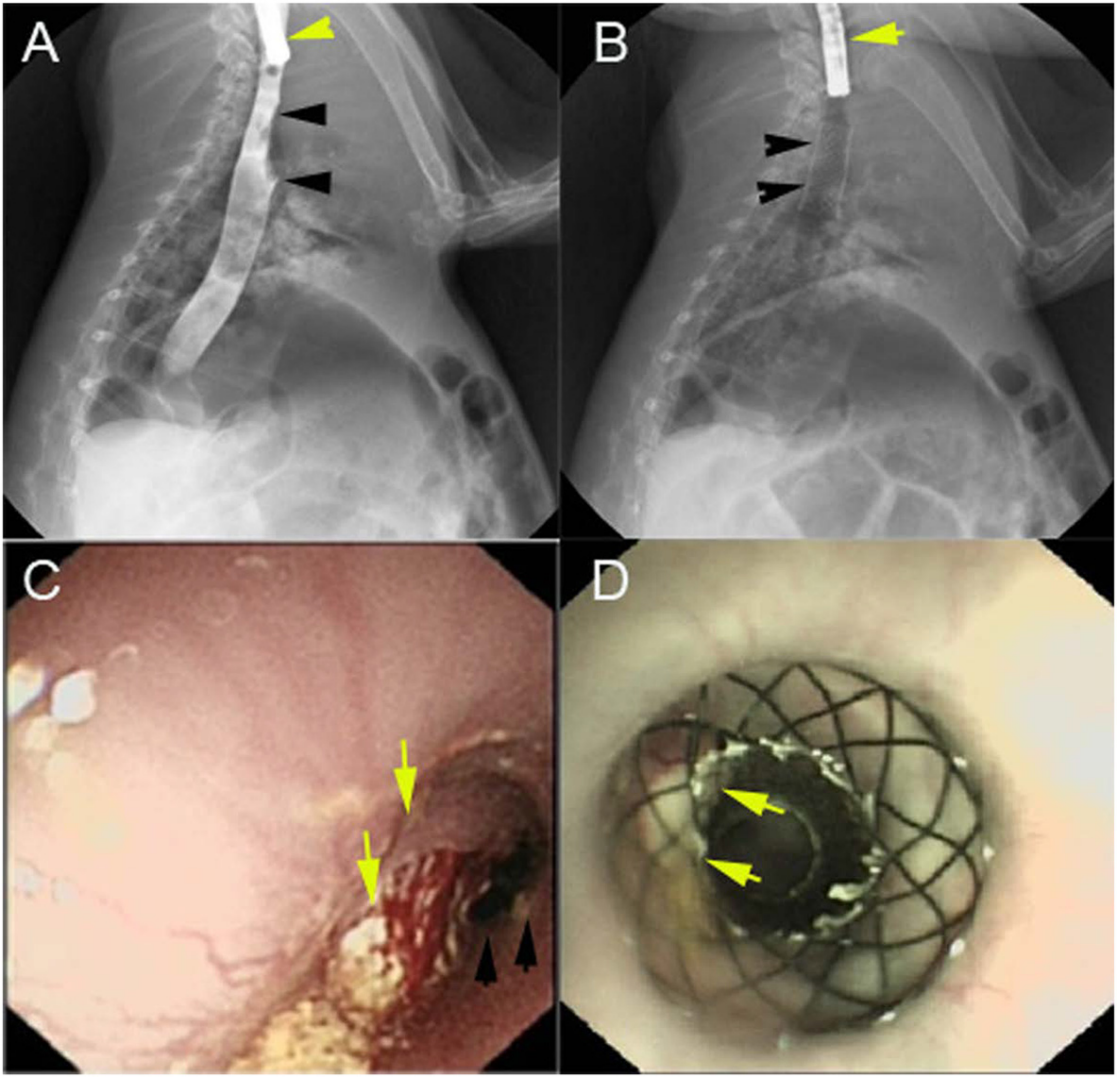
**Procedure for stent placement. (A)** Fluoroscopy showed a malignant stricture (black arrow) in the thoracic esophagus of a rabbit. The endoscope (yellow arrow) was used for injection of the contrast. **(B)** Fluoroscopic view of the stent implanted at the desired location, which was also confirmed using an endoscope (yellow arrow). **(C)** Endoscopic view of the tumor intra-luminal growth (yellow arrow); the esophageal lumen was on the right of the tumor (black arrow). **(D)** After stent placement, the endoscopic view of the stent at an ideal location revealed that the tumor was outside of stent, but within the extension of the stent (yellow arrow).

The stents was randomly implanted in some models when the degree of esophageal stricture became severe, the remainder without stents acted as controls for the survival study. The self-expandable metal stent (8 mm in diameter and 25 mm in length) was designed and manufactured (Garson Company, Changzhou City, China), and its partial length was covered with a silicone membrane (Figure 1). Using X-ray fluoroscopy, a guidewire was first inserted across the stricture into the stomach. Next, the delivery device was gently passed over the guidewire, and the restraining tube was retracted to release the stent within the stricture (Figure 2). As soon as the stent was deployed, correct positioning of the stent was assessed endoscopically. Next, endoscopic and fluoroscopic examinations were performed every week to observe the immediate and late complications of the models, inclouding fistulization/perforation, bleeding, stent migration, esophageal restenosis, airway compromise and procedure-related mortality.

### Survival time

The survival time of all rabbits demonstrated severe esophageal stricture was recorded, independent of the status of stent placement. The rabbits were fed separately in cages to daily observe the quantity of food and water. Paste food or liquid food (vegetable juices) were provided for animals with dysphagia or a 50% reduction of food intake. A humane endpoint was used in this study, in which the animal models were humanely euthanized when they could not have any food or water for several days and their health was extremely weak, such that they could not stand. For the euthanasia procedure, the animal was first anesthetized via an intramuscular injection of 35-mg/kg pentobarbital sodium using the ear venous, and then with a injection of 30 ml air.

### Data analysis

We calculated the success rate of tumor production, inclouding that of endoscopic implantation and tumor development. The rate of severe esophageal stricture and the rate of immediate and late complication of stents placemen were also investigated. The Kaplan-Meier method with the log rank test was used to analyze the survival of rabbits with or without stents. The statistical significance was set at the *P* < 0.05 level. All statistical tests were performed with the SPSS 16.0 software.

## Results

### Success rate of tumor production

**Figure 3:**
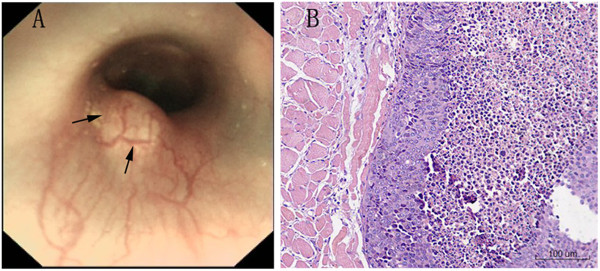
**Tumor development. (A)** Endoscopic view of tumor growth one week after implantation (black arrow). **(B)** Microscopy confirmed the tumor growth.

A total of 38 rabbits were used in this study. Thirty-four rabbits were successfully implanted with VX2 fragments in the esophagus and the success rate of endoscopic implantation was 89.4% (95% CI, 79.6% to 99.2%). Four rabbits were removed from the study because of the esophageal perforations during the endoscopic implantation and two injured rabbits (5.3%) died after conservative treatments. After implantation, tumor development was confirmed (Figure 3) in 30/34 rabbits and the success rate was 88.2% (95% CI, 76.9% to 99.5%).

### Degree of esophageal stenosis

**Figure 4:**
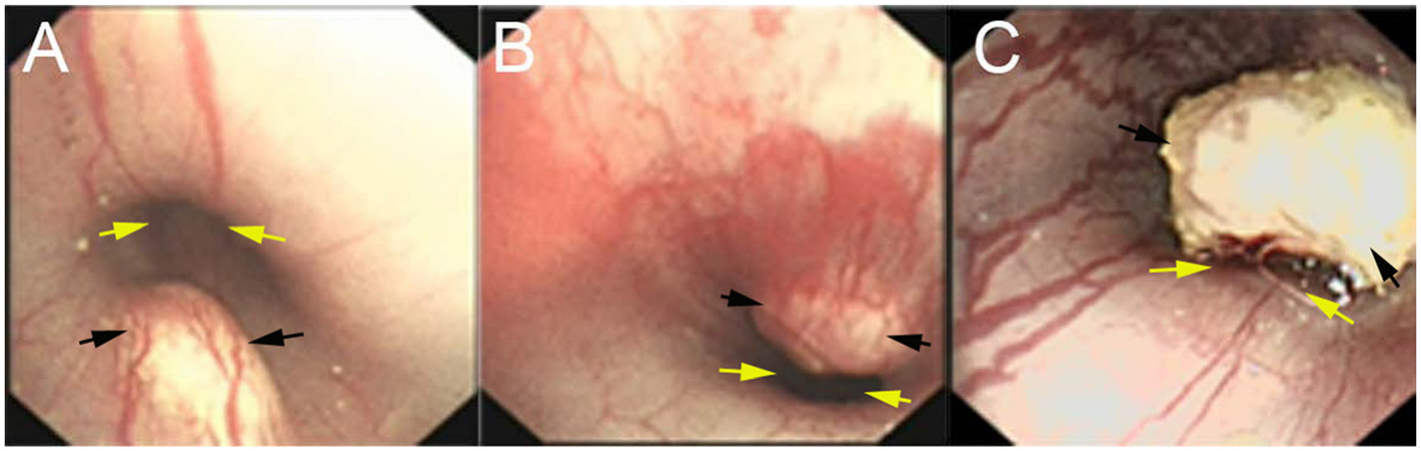
**Degree of esophagostenosis.** The endoscopic follow-up showed **(A)** a mild stricture in a rabbit; **(B)** a moderate stricture in a rabbit; **(C)** a severe stricture in a rabbit. In the image, the black arrows indicate the tumors and the yellow arrows indicate the esophageal lumen.

During the endoscopic follow-up after tumor implantation, severe stricture was observed in 22/30 rabbits (Figure 4), and the rate was 73.3% (95% CI, 57.5% to 89.1%). Mild-to-moderate stricture was observed in 8/30 rabbits and the rate was 26.7% (95% CI, 10.9% to 42.5%).

### Complications of stent placement

**Figure 5:**
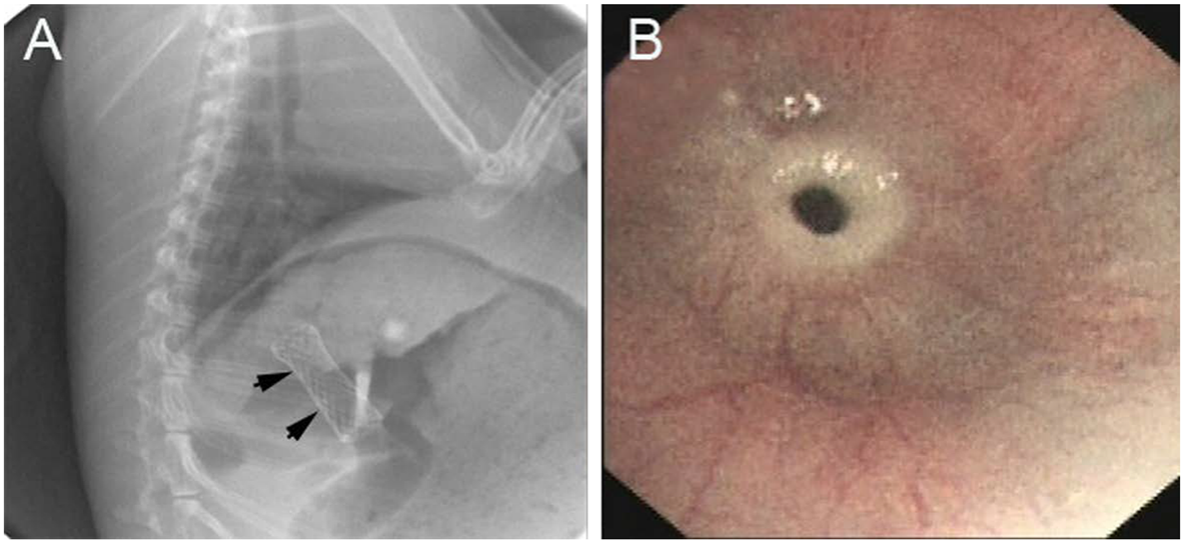
**Complications of stent placement. (A)** During stent implantation, fluoroscopic view of stent migration to the stomach in a rabbit. **(B)** Endoscopic view of the esophageal re-stenosis at the distal stent in an animal model three weeks after stent placement.

Among 22 models with severe esophageal stricture, 12 rabbits were smoothly implanted with esophageal stents, and the remaining 10 rabbits served as controls for the survival study. During and after stent deployment, stent migration, esophageal restenosis (Figure 5) and airway compromise occurred in 2/12 (16.7%), 1/12 (8.3%) and 1/12 (8.3%) rabbits respectively. There was no severe stent-related complication or mortality.

### Survival time of the models

**Figure 6:**
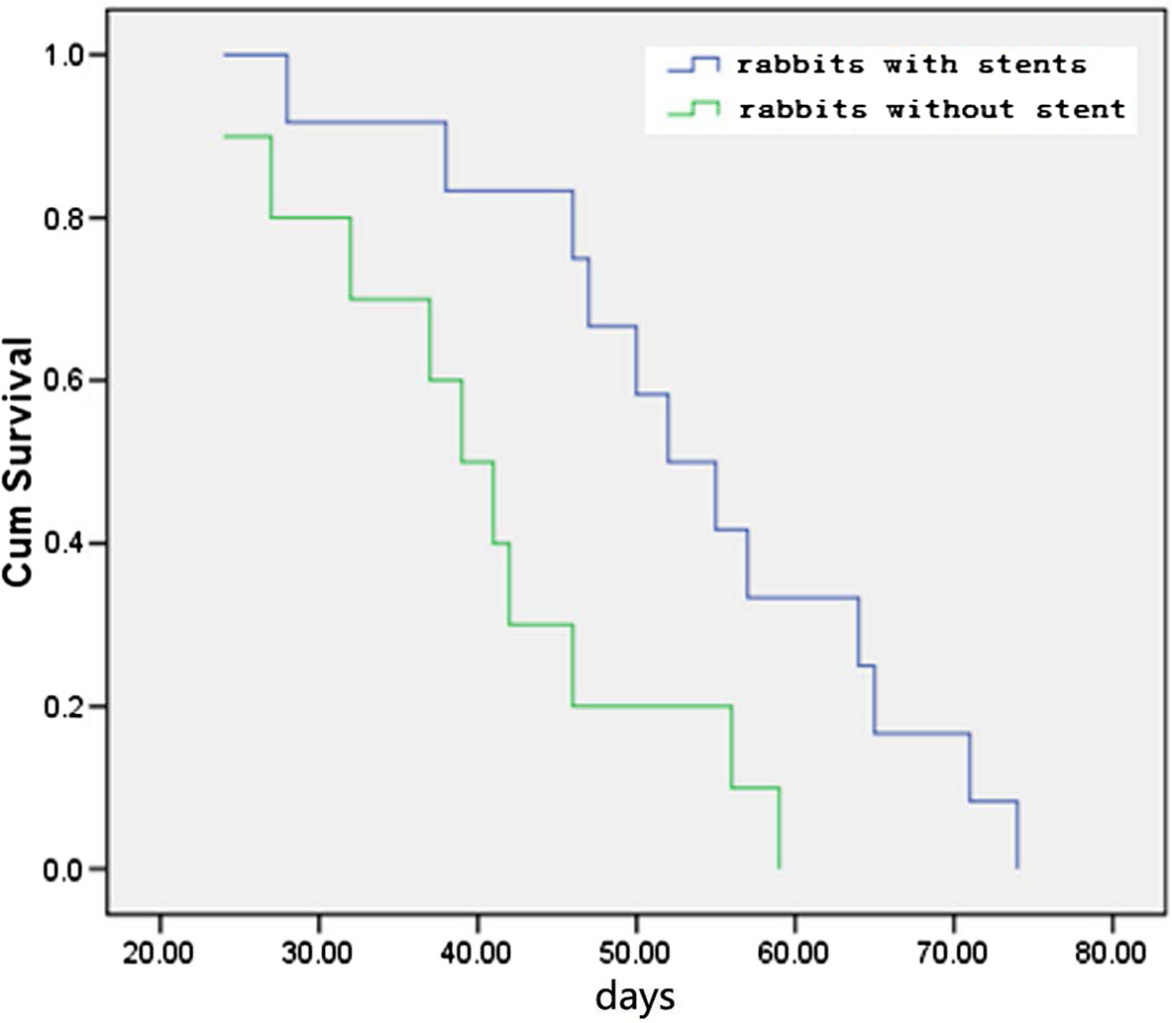
**Survival curve of the rabbits with severe esophagostenosis.** Graph of Kaplan–Meier survival analysis showing a significant survival benefit *(P* < 0.05) for models with stent placement (blue curve), compared to those without stent deployment, but similarly demonstrated severe esophagostenosis (green curve).

The longest survival time of rabbits with stent implantation was 74 days and the shortest survival time was 28 days with a mean survival time of 53.9 ± 13.4 days. However, the longest survival time of rabbits without stents deployed was 59 days, and the shortest survival time was 24 days with a mean survival time of 40.3 ± 11.3 days, which was significantly different (*P* = 0.016) in the survival time between the two groups (Figure 6).

## Discussion

Despite having a higher success rate of tumor growth [15,16] compared to VX2 cells, the VX2 fragments were difficult to implant into the rabbit esophagus, independent of the endoscopic or surgical procedure selected. Compared with the surgical method, the endoscopic procedure was minimally invasive, but it increased the incidence of esophageal perforation when using large needles to implant the VX2 fragments. Thus, we introduced the submucosal injection technique [17,18] for our endoscopic procedure to decrease the complications by enlarging the space of the submucosal layer of the esophagus. In the current study, the endoscopic method demonstrated a high success rate of implantation indicating that it is a safe and effective method for the establishment of a malignant esophagostenosis model.

Esophageal stenosis is a predominant pathological feature of EC in the clinic because it promotes rapid deterioration and death [19,20]. In the present study, our model showed a high rate of severe esophagostenosis, which is a very important indication for stenting. We proposed that this characteristic formation might be associated with the tumor submucosal layer implantation, which contributs to the tumor intra-luminal growth.

In many preclinical studies, large-sized animal models, such as the dog and pig, have been generally used for stenting, but these animals were not under disease conditions, despite the fact that they could simulate the human body environment for esophageal stent deployment. Thus, they were only used to assess the safety of stenting [21-23]. Furthermore, small animal models, such as mice, were immunodeficient and may potentially exhibit malignant esophagostenosis, this, could be used to study the efficiency and mechanism of stenting; however, these small animal models can not allow for stenting, according to the procedures designed for humans [24-26]. Thus, they are unsuitable and are seldom used for studies on stents. The rabbit model of malignant esophagostenosis developed in this study not only exhibits the feature of malignant esophagostenosis, commendably mimicking that of human EC and showing potential use for research on the efficiency and mechanism of stents, but it could also simulate the human body environment for stenting according to the procedures used for humans.

The overall complication rate and primary success rate of self-expanding esophageal stenting in clinical research were approximately 30% and 97.2%, respectively [27,28]. In this study, all of the procedures of the stent implantation were smoothly completed, and there was no rabbit that had severe stent-related complications [29,30], such as fistulization/perforation or bleeding, and there was no procedure-related mortality, although the events of stent migration, esophageal re-stenosis and airway compromise occurred with a total complication rate of 33.3% during and after stent implantation. These results indicated that it is safe and feasible to implant an esophageal stent into our animal models. Moreover, the survival time of rabbits with stent placement was longer compared to rabbits without stent deployment indicating that our models could endure stent implantation and benefit from the stents. This finding suggested that our animal model represents an ideal tool for the study of esophageal stents for EC.

The limitation of our animal model is that it is an orthotopic allograft tumor model and cannot be maintained the routine course of esophageal cancers. This study focused on whether the animal model is suitable for stent implantation and may neglect other features and utility of this animal model.

In conclusion, the endoscopic method is safe and effective for the development of a rabbit model of malignant esophagostenosis to mimic the progression of this human disease. This model can simulate human the body environment for stent deployment and is an excellent tool for studying stent innovation for the treatment of esophageal cancer.

## Competing interests

The authors declare that they do not have any competing or financial interests.

## Authors’ contributions

ZF, JH, JS, XW and GX conceived, designed and performed the experiments. YZ, XT, YS, HS and ZL analyzed the data and wrote the paper. All authors read and approved the final manuscript.

## Supplementary Material

Additional file 1: Endoscopic method for implantation of VX2 tumors. Click here for file
